# Grazing weakens temporal stabilizing effects of diversity in the Eurasian steppe

**DOI:** 10.1002/ece3.3669

**Published:** 2017-11-26

**Authors:** Haiyan Ren, Friedhelm Taube, Claudia Stein, Yingjun Zhang, Yongfei Bai, Shuijin Hu

**Affiliations:** ^1^ College of Agro‐grassland Science College of Prataculture Science Nanjing Agricultural University Nanjing China; ^2^ Institute of Crop Science and Plant Breeding‐Grass and Forage Science Christian‐Albrechts‐University Kiel Germany; ^3^ Tyson Research Center and Department of Biology Washington University St. Louis St. Louis MO USA; ^4^ Department of Grassland Science China Agricultural University Beijing China; ^5^ State Key Laboratory of Vegetation and Environmental Change Institute of Botany Chinese Academy of Sciences Beijing China; ^6^ College of Resources and Environmental Sciences Nanjing Agricultural University Nanjing China; ^7^ Department of Entomology and Plant Pathology North Carolina State University Raleigh NC USA

**Keywords:** diversity–stability relationship, grazing intensity, plant diversity, plant functional groups, semi‐arid steppe, species asynchrony, temporal stability

## Abstract

Many biodiversity experiments have demonstrated that plant diversity can stabilize productivity in experimental grasslands. However, less is known about how diversity–stability relationships are mediated by grazing. Grazing is known for causing species losses, but its effects on plant functional groups (PFGs) composition and species asynchrony, which are closely correlated with ecosystem stability, remain unclear. We conducted a six‐year grazing experiment in a semi‐arid steppe, using seven levels of grazing intensity (0, 1.5, 3.0, 4.5, 6.0, 7.5, and 9.0 sheep per hectare) and two grazing systems (i.e., a traditional, continuous grazing system during the growing period (TGS), and a mixed one rotating grazing and mowing annually (MGS)), to examine the effects of grazing system and grazing intensity on the abundance and composition of PFGs and diversity–stability relationships. Ecosystem stability was similar between mixed and continuous grazing treatments. However, within the two grazing systems, stability was maintained through different pathways, that is, along with grazing intensity, persistence biomass variations in MGS, and compensatory interactions of PFGs in their biomass variations in TGS. Ecosystem temporal stability was not decreased by species loss but rather remain unchanged by the strong compensatory effects between PFGs, or a higher grazing‐induced decrease in species asynchrony at higher diversity, and a higher grazing‐induced increase in the temporal variation of productivity in diverse communities. Ecosystem stability of aboveground net primary production was not related to species richness in both grazing systems. High grazing intensity weakened the temporal stabilizing effects of diversity in this semi‐arid grassland. Our results demonstrate that the productivity of dominant PFGs is more important than species richness for maximizing stability in this system. This study distinguishes grazing intensity and grazing system from diversity effects on the temporal stability, highlighting the need to better understand how grazing regulates ecosystem stability, plant diversity, and their synergic relationships.

## 1 INTRODUCTION

The effects of biodiversity on stability of ecosystem functioning have received much attention because of accelerated species loss and the associated risks for the stability of ecosystem and the reliable provisioning of ecological services (Baert, De Laender, Sabbe, & Janssen, 2016; Hautier et al., 2014, 2015). Many biodiversity experiments have shown that greater species diversity promotes greater temporal stability of aboveground net primary production (ANPP; Gross et al., 2014; Jiang & Pu, 2009; Pimm, Russell, Gittleman, & Brooks, 1995). One major underlying mechanism is that species asynchrony is a major driver of ecosystem stability, where can be driven by competitive interactions and environmental responses (Hooper & Vitousek, 1997; Isbell, Polley, & Wilsey, 2009; Xu et al., 2015). The more diverse communities are the more likely they are to be a decrease in the biomass of some species and an increase in others (Tredennick, Adler, & Adler, 2017). By influencing the degree of species asynchrony via regulating species’ diverse responses to grazing, grazing may also decrease the temporal stability of ANPP (Hautier et al., 2014, 2015; Loreau & de Mazancourt, 2013). The stability of ecological systems is directly lessened by anthropogenic land use, which could trigger species extinctions and reductions in ecosystem services (Hautier et al., 2014, 2015; Xu et al., 2015). Understanding which factors driver the stability is essential for managing the long‐term sustainability of plant productivity and animal productivity in grassland ecosystem.

In semi‐arid and arid ecosystems, unsustainable livestock farming is considered the biggest driver of ecological stability reduction and diversity loss in many parts of the world. Grazing system and grazing intensity strongly affect plant community structure and ecosystem functioning (e.g., nutrient cycle, plant productivity; Hallett, Stein, & Suding, 2017; Hickman, Hartnett, Cochran, & Owensby, 2004; Hoffmann et al., 2016). Different types of grazing systems (e.g., rotational vs. continuous) may impact ecosystem stability because each system results in unique temporal and spatial environments that differentially affect species’ dynamics. In comparing with continuous grazing system, grazing press in mixed grazing system was mitigated by removing aboveground biomass without trampling and excretion in alternate years, and has higher resilience and lower grazing resistance, and thus assumes to have higher stability. As known, the plant functional groups (PFGs) of dominant perennial rhizomatous grasses and perennial bunchgrasses have strong compensation capabilities to switch dominance (Pan et al., 2016). That is the increase or decrease of rhizomatous grasses always accompanies contrary variations of bunchgrasses. Dominant PFGs are assumed to depress their compensation capabilities under higher grazing intensity (rhizomatous grasses should be fairly resistant to grazing while bunchgrasses should be impacted greater), and further change diversity–stability relationships. How different grazing system could modulate PFGs’ compensatory effects and further affect ecosystem stability in grazed grasslands remain unknown. Moreover, grazing intensity could either positively or negatively affect species diversity (Grace et al., 2007; Hickman et al., 2004; Li, Xu, Zheng, Taube, & Bai, 2016). Existing diversity‐disturbance models and hypotheses suggest that intermediate levels of disturbance lead to peak diversity in grazed communities, resulting from a combination of disturbance‐tolerant species and competitive interactions under moderate grazing (Beck, Hernandez, Pasari, & Zavaleta, 2015; Cingolani, Noy‐Meir, & Diaz, 2005; Kondoh & Williams, 2001). According to a further refinement of MSL (Milchunas, Sala, and Lauenroth) model (Milchunas et al. 1988, Cingolani et al., 2005; Schonbach et al. 2011), diversity–grazing intensity relationship correspondingly fit to one typical equilibrium curves of four generalized models (increasing, decreasing, unimodal, or no response) for grasslands, depending on environmental moisture, community productivity, and evolutionary history of grazing. Correspondingly, these models can be used to set up predictions for how grazing affects the diversity–ecosystem stability relationship. In current study region, the semi‐arid grassland was considered as low‐productivity systems owing to low ANPP (under 200 DM g/m^2^) and low annual precipitation (330 mm) and had a long‐term grazing history (more than 20 years) in the context of the MSL model, and thus fit to decreasing curves for species diversity–grazing intensity relationship. Whether the ecosystem stability–grazing intensity relationship also fits to the decreasing equilibrium curves following MSL model, remains unknown. As species diversity is considered to be positively related to ecosystem stability (Gross et al., 2014; Jiang & Pu, 2009), we expect that high grazing intensity decreases the stability of ANPP along with directly changing species diversity in our grazing system. Combining species response and species diversity variation, how grazing system and grazing intensity and their interaction effects on grassland stability of ANPP need to be further explored.

Rather than directly changing species diversity, grazing could also exert additional impacts on ecosystem stability by interacting with climate factors, for example, precipitation. In natural grasslands, high grazing intensity changes species’ functional traits and their responses to environmental fluctuations (Li et al., 2016), which may in turn affect the degree of species asynchrony, and thus the temporal stability of ANPP. However, species diversity is likely invariant under the interactions of precipitation and grazing (Gossner et al., 2016; Hooper et al., 2012). For instance, overgrazing may limit species richness, but high precipitation could enhance species richness and thus offset its negative effects on species diversity. Moreover, grazing could serve to depress stability by reducing the temporal mean of productivity or enhancing its standard deviation or both instead of decreasing species diversity (Hautier et al., 2014, 2015). Ecosystem stability includes resilience and resistance, and the temporal stability of ANPP is defined as the ratio of the temporal mean to its temporal variability, that is, the temporal standard deviation over a period. As ANPP is strongly affected by grazing intensity, grazing may affect stability by changing the temporal mean of ANPP at different intensity levels; but grazing effects on temporal standard deviation of ANPP are unknown. Hautier et al. (2014) evaluated the species diversity–stability of ANPP relationships in natural and fertilized sites including 41 naturally assembled grasslands. However, besides nutrient availability, grazing management strategies including the grazing intensity and the grazing system, which have important consequences for ecosystem functioning, also need to be considered. The findings could offer a sound basis for designing grazing management strategies that optimize productivity, sustaining ecosystem stability in semi‐arid grasslands.

We used a six‐year grazing experiment in the Inner Mongolian grassland which is the representative of the Eurasian steppe (Bai et al., 2007), to analyze ecosystem stability in response to different grazing management practices. We tested how diversity–stability of ANPP relationships changed along a grazing intensity gradient within a traditional continuously grazed and a mixed grazed/mowed system. The study was conducted to test (1) PFGs slightly respond to grazing intensity in MGS than in TGS, and further lead different diversity–ecosystem stability relationships in two grazing systems; and (2) high grazing intensity decrease temporal stability relationships according to MLS model.

## 2 MATERIALS AND METHODS

### 2.1 Study area

The experiment was located in the semi‐arid grassland of the Xilin River basin, near the Inner Mongolia Grassland Ecosystem Research Station (IMGERS, 43°38′ N, 116°42′ E, located at about 1,200 m a.s.l.) of the Chinese Academy of Sciences in P.R. China (Bai, Han, Wu, Chen, & Li, 2004). The average annual temperature in the region is 0.9°C (1982–2010). Mean annual precipitation is 329 mm (1982–2010), with more than 70% of annual precipitation occurring during the growing season from April to September (Ren, Schoenbach, Wan, Gierus, & Taube, 2012; Figure S1). The predominant soil types of this region are calcic chernozems (IUSS Working Group WRB 2006), which cover acid volcanic parent rock. The grazing history of the study site (160 ha) in the past 15 years prior to the start of our experiment averaged 10–12 sheep per hectare and represents medium to heavy grazing intensity (Reszkowska et al. 2011). To allow the grass sward to recover from grazing and to ensure comparable grass availability among treatments, grazing was stopped 2 years before the start of the experimental treatments, and in August, the site was uniformly cut. The investigated typical steppe ecosystem consisted of about 36 plant species, which can be classified into four PFGs: perennial rhizomatous grasses, perennial bunchgrasses, perennial forbs, and annuals/biennial grasses (Sasaki et al., 2009; Wu et al., 2015).The two most abundant species are the perennial rhizomatous grass *Leymus chinensis*, and the perennial bunchgrass *Stipa grandis*, which together account for about 75% of total aboveground biomass (Li et al., 2016).

### 2.2 Experimental design

In 2005, we established a 6‐year grazing experiment in two replicated blocks differing by topographic position (one flat block and one sloping block on slopes with inclinations of <10°; Hoffmann, Funk, Li, & Sommer, 2008). In each block, nine replicates were applied of seven randomized placed grazing intensity treatments. In a randomized split‐plot design, we established at the main plot level two management systems (i.e., traditional continuous grazing versus mixed grazing system). The traditional continuous grazing system (TGS) involved annual grazing during the vegetation period (June‐September). The mixed grazing system (MGS) involved annual alternation between grazing and mowing (simulating grazing for removal of plant shoot tissue monthly). Within each management system, we established 2 ha (enlarge to 4 ha at the treatment of lower grazing intensity = 1.5 sheep/ha for a guarantee of six sheep per plot at least) plots that received one of seven levels of grazing intensity treatments (i.e., stocking rates ranged from 0, 1.5, 3.0, 4.5, 6.0, 7.5, 9.0 sheep/ha). Starting from June to the end of mid‐September (i.e., most of the growing season), nonlactating 15‐month‐old female sheep with an average live weight of 35 kg was used for grazing.

### 2.3 Measurements

To estimate grazing effects on aboveground net primary productivity (ANPP), three 2 m × 3 m exclosure cages in each plot were set up and moved monthly during the growing season. Peak aboveground biomass was measured as ANPP in the ungrazed control plots. Aboveground biomass inside and outside the exclosure cages was measured each month to estimate ANPP in the grazed (G) plots (Li et al., 2016; Schonbach et al., 2011). The equation of total ANPP in grazed plots is as follows:

(1) $${\text{ANPP}}_{G}=\;\text{W1g}\;+\;\left(\text{W2u}\;-\;\text{W1g}\right)\;+\;\left(\text{W3u}\;-\;\text{W2g}\right)\;+\;\left(\text{W4u}\;-\;\text{W3g}\right),$$

where W_i_ is the aboveground biomass at sampling time *t*_i_ (i = 1, 2, 3, 4: beginning of June, July, August, and September, respectively). Indices u (ungrazed) and g (grazed) mean samplings inside and outside the exclosure cages.

Plant aboveground biomass was clipped to 1 cm stubble height inside and outside of each exclosure cage, sorted by species, dried at 60°C to a constant mass and then weighed. Inside and outside of each exclosure cage, species composition, and species richness which defined as the number of species in each plot were assessed by applying a conventional quadrat sampling procedure in the sampling units (0.25 × 2 m; Whalley & Hardy, 2000). In this study, nine replicates (sample size around 2 ha) from each grazing intensity at each block within two systems were used. To test the effects of grazing on stability of ANPP, species richness and productivity over the sampling period (2005–2010), we calculated the mean aboveground biomass (μ), its standard deviation (σ), and their ratio over the 6 years as measures of temporal stability of community ANPP (μ/σ). Ecosystem stability has been measured in various ways in previous ecological studies (Hautier et al., 2014, 2015; Tilman, Reich, & Knops, 2006). Here, we use the temporal stability of ANPP (μ/σ). The temporal mean of species dominance and community grazing resistance were all analyzed.

(2) $$\text{Resistance}=\ln\left(\frac{{\text{biomass}}_{\mathrm{Gi}}}{{\text{biomass}}_{G0}}\right)$$

where resistance refers to the relative rate of plant community biomass change along the grazing gradient, and biomass_Gi_ (i = 0, 1.5, 3, 4.5, 6, 7.5, 9 sheep/ha from very lightly grazing to heavily grazing intensity) and biomass_G0_ are the aboveground biomass at different grazing intensity levels (1 = 1.5, 3, 4.5, 6, 7.5, 9 sheep/ha) and in the no grazing plots, respectively. Values close to 0 imply greater resistance and less change in biomass due to grazing.

Species asynchrony, which is used to compare stability among communities with diverse species numbers and PFGs (Hautier et al., 2014; Isbell et al., 2009), was identified by following equation:

(3) $$\text{Species}\;\text{asynchrony}=\frac{1-\sigma^{2}}{{\left(\sum \sigma_{i}\right)}^{2}}$$

where σ^2^ is the temporal variation of community ANPP, σ_i_ is the standard deviation of aboveground biomass of species i over 6 years.

### 2.4 Statistical analysis

To assess the effects of grazing system, grazing intensity and their interactions on all related variables, repeated measures analysis of variances (RMANOVA) processed with a mixed model were performed using the grazing system, grazing intensity treatments as the between subject factors and the year as a within‐subject factor (Table S1). To address the diversity–grazing intensity relationship, least squares methods with adjusted *R* ^2^ and lowest corrected Akaike information criterion (AIC) were used to perform curve fitting. By goodness‐of‐fit tests for different regression models, nonlinear multivariate regression model was used to show PFGs response to grazing intensity in two grazing system; quadratic regressions as MSL model with best‐adjusted *R* ^2^ were selected for addressing our second hypothesis linked to grazing–ecosystem stability relationships and diversity–ecosystem stability relationships in grazed grassland. The quadratic regression analysis of aboveground biomass of the two primary PFGs (bunchgrass and rhizomatous grass), temporal mean/standard deviation/stability of ANPP, species asynchrony, and richness along with grazing intensities was all tested by goodness‐of‐fit of linear model with higher adjusted *R* ^2^. The level of significance was *p* < .05. The quadratic regressions as MSL model were also used to test the correlation of species richness and temporal mean/standard deviation/stability of ANPP and species asynchrony. SAS Version 9.1 (SAS Institute Inc., Cary, NC, USA) was used for all analyses and plotting.

## 3 RESULTS

### 3.1 Grazing effects on ANPP, species richness of PFGs, and temporal stability

**Figure 1:**
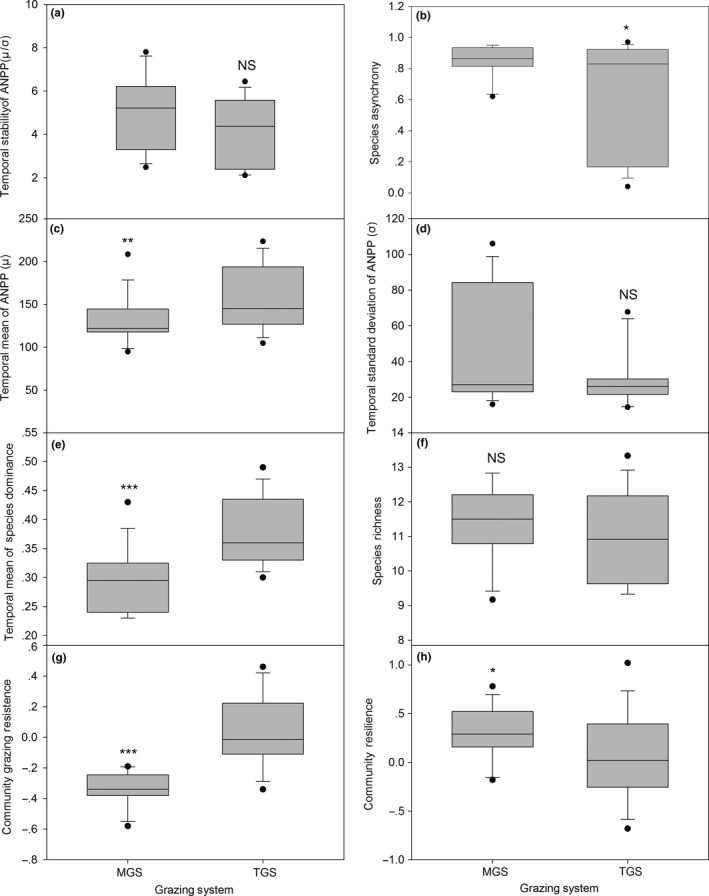
Effects of a mixed grazing system (MGS) and traditional grazing system (TGS) on (a) ecosystem stability of aboveground net primary production (ANPP), (b) species asynchrony, (c) temporal mean of ANPP, (d) temporal standard deviation of ANPP, (e) temporal mean of species dominance, (f) species richness, (g) community grazing resistance, and (h) community resilience in semi‐arid steppe in Inner Mongolia over a period of six years. (*: .01 < *p *<* *.05, **: .001 < *p *<* *.01, ***: *p *<* *.001, and NS indicates not significant, *p *>* *.05). Values are means ± 1*SE*

**Figure 2:**
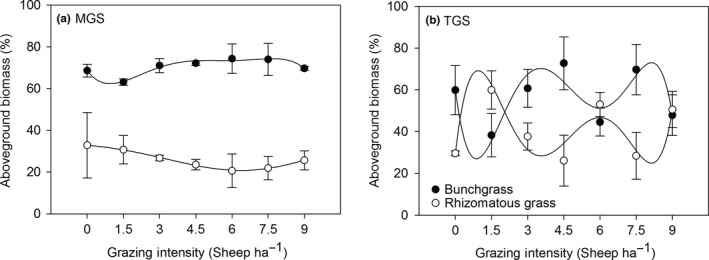
Aboveground biomass (%) of dominant plant functional groups in the whole grassland plant communities during the experimental period from 2005 to 2010 in a mixed grazing system (a) and a traditional grazing system (b; *N* = 14) along a grazing intensity (GI) gradient (GI = 0, 1.5, 3, 4.5, 6.0, 7.5, 9.0, from ungrazed to heavy grazed) in semi‐arid steppe in Inner Mongolia. Nonlinear multivariate regression model was used to smooth the scatter plot

**Figure 3:**
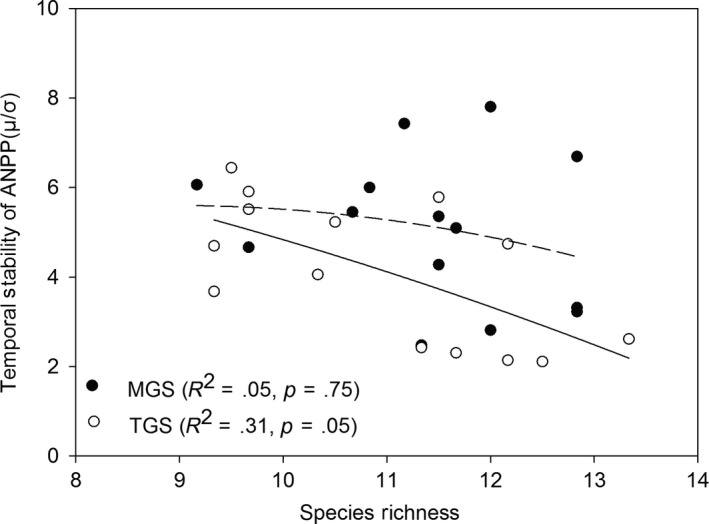
Relationships of the community stability of aboveground net primary production and species richness in a mixed grazing system (MGS) and a traditional grazing system (TGS). Quadratic regressions were used to smooth the scatter plot and highlight their relationship, although the statistical result was not significant

The repeated‐measure analysis of variance (RMANOVA) showed that the effects of grazing system on the ANPP percentage of all four PFGs in the whole plant communities were highly significant (Table S1). ANPP was 20% higher in TGS than in MGS. The interactions of grazing system and grazing intensity as well as grazing intensity itself had no significant effect on either ANPP or richness of all PFGs in the community (except for ANPP of bunchgrasses and richness of annual/biennial grasses). ANPP and richness of PFGs were all affected by years (*p* ≤ .001). The year effects were attributed to the annual variation in climate factors, including mainly precipitation and temperature (Figure S1). The correlation between ANPP and precipitation and temperature in this study area has been confirmed (Ren et al., 2012; Zheng, Li, Lan, Ren, & Wang, 2015). For all PFGs examined, the major factors influencing ANPP and richness were grazing system and year. There was no significant difference in temporal stability of ANPP, temporal standard deviation of ANPP, and species richness between the two systems (Figure 1a,d,f), but TGS had lower species asynchrony (Figure 1b). The TGS had higher temporal mean ANPP and species dominance compared to the MGS (Figure 1c,e). From 2005 to 2010 year, ANPP in TGS along with grazing intensity was coincidently higher than it in MGS was, and had greater variations in response to grazing (Figure S3). The dominant species made a greater contribution to temporal ANPP in TGS over 31% species dominance than MGS. Grazing resistance of plant community over time was much greater in TGS than in MGS (Figure 1g), but inversely in community resilience (Figure 1h). The aboveground biomass of dominant PFGs (bunchgrass and rhizomatous grass) in MGS was relatively consistent across grazing intensities but showed compensatory variations in TGS, with a decrease in the aboveground biomass of bunchgrass compensated by an increase of rhizomatous grasses under each grazing intensity (Figure 2). Four PFG's richness percentage along with grazing intensity was shown in Figure S2. Both MGS and TGS presented a negative but not significant richness–stability relationship, suggesting that a higher diversity community is inclined to lower stability of ANPP in grazed grassland (*R* ^2^_MGS_ = .05, *p* = .052; *R* ^2^_TGS_ = .31, *p* = .748; Figure 3).

### 3.2 The relationship between species richness, species asynchrony, and temporal stability under grazing

**Figure 4:**
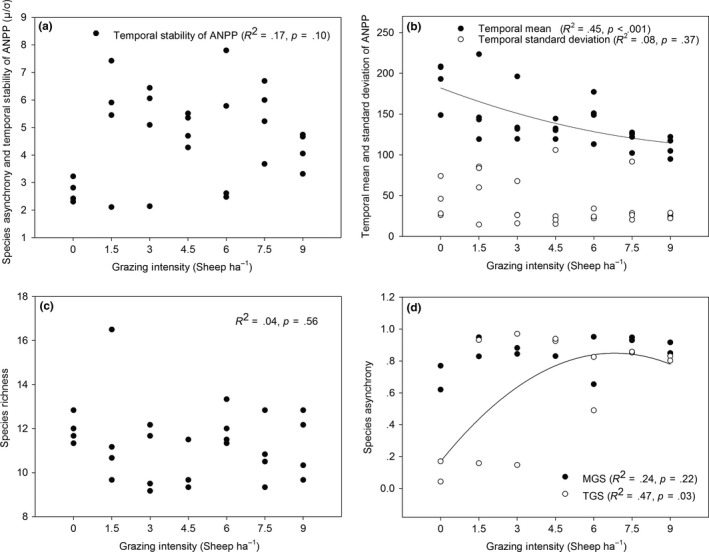
Grazing intensity in relation to (a) ecosystem stability of aboveground net primary production (ANPP;* R* ^2^_stability_ = .17, *p* = .10); (b) temporal mean and temporal standard deviation of ANPP (*R* ^2^_mean_ = .45, *p* < .05; *R* ^2^_*SD*_ = .08, *p* = .37); (c) species richness (*R* ^2^ = .04, *p* = .56); (d) species asynchrony in a mixed grazing system (MGS) and a traditional grazing system (TGS;* R* ^2^_MGS_ = .24, *p* = .22; *R* ^2^ _TGS_ = .47, *p* = .03). Quadratic regressions with significant relationships were used to smooth the scatter plot

As ANOVA showed that there was no interaction between grazing intensity and grazing system. The averaged effects of grazing intensity across both grazing systems were used in the following analyses of temporal stability (Table S1). Temporal stability of ANPP did not significantly change with grazing intensity (*R* ^2^_stability_ = .17, *p* = .10; Figure 4a). The temporal mean of ANPP decreased along with grazing intensity (*R* ^2^ = .45, *p* < .001), but its standard deviation had no significant response (*R* ^2^ = .08, *p* = .37; Figure 4b). Species richness was also not significantly affected by grazing intensity (*R* ^2^ = .04, *p* = .56; Figure 4c). The positive relationship between grazing intensity and species asynchrony in TGS showed that higher grazing intensity enhanced species asynchrony (*R* ^2^ = .47, *p* = .03) within TGS, but not in MGS (*R* ^2^ = .24, *p* = .22; Figure 4d).

**Figure 5:**
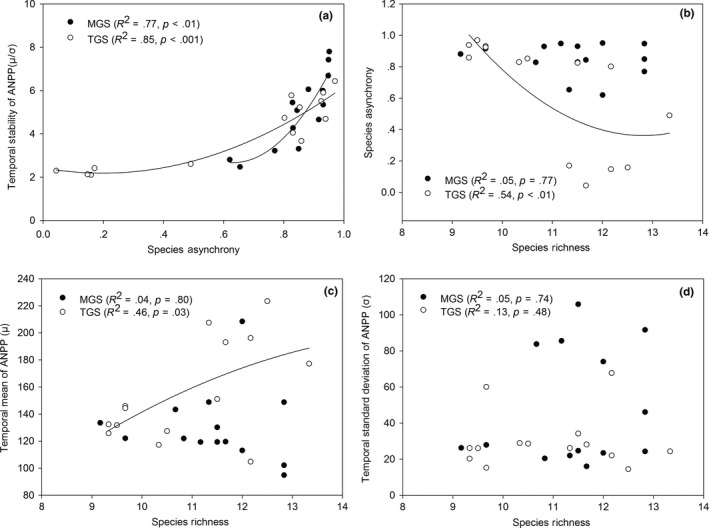
(a) Relationship of ecosystem stability of aboveground net primary production (ANPP) with species asynchrony (*R* ^2^ _MGS_ = .77, *p* < .01; *R* ^2^ _TGS_ = .85, *p* < .001); (b) species richness in relation to species asynchrony (*R* ^2^ _MGS_ = .05, *p* = .77; *R* ^2^ _TGS_ = .54, *p* < .01); (c) species richness in relation to temporal mean (*R* ^2^ _MGS_ = .04, *p* = .80; *R* ^2^ _TGS_ = .46, *p* = .03); and (d) species richness in relation to temporal standard deviation of ANPP (*R* ^2^ _MGS_ = .05, *p* = .74; *R* ^2^ _TGS_ = .13, *p* = .48) in a mixed grazing system (MGS) and a traditional grazing system (TGS)

To evaluate the effects of grazing treatments on the relationships among target variables in two grazing system, multiple regressions between related variables were used here (Figure 5). Regression analyses showed that species asynchrony was positively correlated with ecosystem stability of ANPP in grazed communities in both grazing system (*R* ^2^_MGS_ = .77, *p* < .01; *R* ^2^_TGS_ = .85, *p* < .001; Figure 5a). Species richness was negatively correlated with species asynchrony under TGS (Figure 5b, *R* ^2^_TGS_ = .54, *p* < .01). When comparing the relationships between species richness and temporal mean or standard deviation of ANPP in grazed grassland, we found that temporal mean of ANPP was positively related to species richness in TGS (*R* ^2^_TGS_ = .46, *p* < .05) but not in MGS (*R* ^2^_MGS_ = .04, *p* = .80; Figure 5c). The standard deviation of ANPP was not affected by species richness in both grazing systems (*R* ^2^_MGS_ = .05, *p* = .74; *R* ^2^_TGS_ = .13, *p* = .48; Figure 5d), which indicated that grazing reduced the negative effect of species richness on the temporal standard deviation of ANPP. Thus, grazing could decrease the temporal stability of ANPP in highly diverse communities, as a result of an increase in the temporal standard deviation in diverse communities.

## 4 DISCUSSION

Our results demonstrate the importance of continuous grazing system and high grazing intensity in reducing the temporal stability of ANPP in a steppe ecosystem in Inner Mongolia and contribute to our understanding of the potential mechanisms. In our study, grazing system and grazing intensity did not directly drive variation in ecosystem temporal stability, but resulted in a change in the relationship among species richness, temporal stability, and productivity. The mixed grazing system did not show a higher temporal stability of ANPP and species richness, even though grazing was mitigated by removing aboveground biomass without trampling and excretion in alternate years. The higher community productivity, species dominance, and community grazing resistance in the traditional grazing system indicated that the dominant PFGs contributed more to ANPP in the continuously grazed grasslands, and enhanced community resistance overtime. Previous studies have also reported the key role of dominant species in modulating ecosystem stability (Smith & Knapp, 2003; Wilsey & Martin, 2015).

In both grazing management systems, the temporal stability of ANPP was not significantly influenced by grazing intensity. However, the ways in which they maintained system stability differed, which was related to internal shifts in the biomass balance between dominant PFGs: (i.e., bunchgrass and rhizomatous grasses). The biomass of these two PFGs was persistent along with grazing intensity in the mixed grazing system, but there was a compensatory interaction under each grazing intensity in the traditional grazing system, where the decrease or increase of bunchgrass biomass at each grazing intensity was compensated by an increase or decrease of rhizomatous grasses. As it has confirmed that there was no significant difference either in species composition or species relative abundance before the treatment was conducted (Li et al., 2016), the variations of PFGs along with grazing intensity in two systems should attribute to their compensation effects. A recent study in an adjacent experimental location has shown that these two dominant PFGs and their interactions strongly effect the ecosystem, explaining 42%–77% of the variation of examined ecosystem functions (Pan et al., 2016). Species removal experiments have indicated that dominant PFGs can mitigate the losses of other functional groups, and thus, the impacts of species diversity may be insignificant (Grace et al., 2007; Pan et al., 2016; Winfree, Fox, Williams, Reilly, & Cariveau, 2015). Dominant species or PFGs, rather than rare ones, play an important role in sustaining ecosystem functions and biodiversity (Longo, Seidler, Garibaldi, Tognetti, & Chaneton, 2013; Winfree et al., 2015). Dominant PFGs in the community contribute disproportionately to productivity and abundance and therefore dominate ecosystem properties, as suggested in the mass ratio hypothesis (Grime, 1998; Sandau et al., 2016; Smith & Knapp, 2003). Grazing did not lead to persistence of two or more PFGs overlapping under any grazing intensity, which is attributed to the life history and biological functional traits of various PFGs (e.g., grazing resilience abilities and regrowth rates; Zheng et al., 2015, 2010). Palatable rhizomatous grasses in the study area are rarely susceptible to grazing effects, owing to their strong compensatory capability for rapid regrowth and well‐developed rhizome system (Wang, Li, Han, & Ming, 2004). The native bunchgrasses also can persist under high grazing intensities because of their perennial life cycle and unpalatability (Hamilton, Holzapfel, & Mahall, 1999). Ecosystem stability may rely on constantly regulating internal PFG composition to maintain functional stability in continuous grazing systems. The well‐developed compensatory capability between PFGs may mitigate their negative response to grazing intensity influences, reduce variability in biomass production, and thus maintain dynamic stability of the grazed grassland ecosystem in the short term.

Temporal stability of community ANPP did not respond to change in grazing intensity and grazing system, which seems to be suggestive of grazing‐independent stability. However, the grazing intensity–species asynchrony regression showed that higher grazing intensity enhanced species asynchronous responses in continuous grazing system. Grazing intensity decreased the temporal mean of ANPP but did not affect species richness. This is consistent with the conclusion that productivity does not necessarily depend on diversity (Adler et al., 2011; Grace et al., 2016; Waide et al., 1999). Generally, positive diversity‐stability patterns have been observed in experimentally manipulated communities (Cardinale et al., 2006; Gross et al., 2014). However, in natural grasslands, there is no consistent diversity–stability relationship (Adler et al., 2011). Other ecological factors, such as climate change and limiting nutrient resources, have been identified as more critical drivers (Hautier et al., 2014, 2015; Xu et al., 2015). Our findings agree with those of Stein, Harpole, and Suding (2016) who found that in overgrazed areas reduction in grazing intensity alone may not help recover the system. Our study found that grazing alleviated the positive relationship between species richness and community stability in both grazing systems.

Species asynchrony, driven by unique responses to species to environmental conditions, is an important mechanism for understanding ecosystem stability (Hautier, Niklaus, & Hector, 2009; Tilman et al., 1997). Our analyses showed that grazing did not change the positive species asynchrony–ecosystem stability relationship but led to a negative species richness–species asynchrony relationship in continuous grazing system, which did not support the commonly positive richness‐species asynchrony relationship in 41 natural grasslands (Hautier et al., 2014). Grazing converted the positive effect of species diversity on species asynchrony and stability. We originally expected that according to MSL model higher grazing would decrease species diversity and thus stability in our grazing systems. However, our study found that species richness and temporal stability did not respond to changes in grazing. A decrease in ecosystem stability could also result from a decrease in the temporal mean of ANPP with species diversity or an increase in the temporal variation of ANPP with species diversity, or both. A performance‐enhancing effect supported higher ecosystem stability at higher diversity, which is attributed to a higher temporal mean of ANPP at higher diversity (Hooper & Vitousek, 1997; Tilman et al., 1997). However, in mixed grazing system, the temporal mean of ANPP was not related to species richness.

In comparison with natural grasslands from Hautier et al. (2014)'s results, this study suggests an increase in the temporal variation of ANPP with species richness. In other words, the supposed diversity‐dependent stability actually resulted from a diversity‐related increase in the standard deviation of ANPP relative to its mean under grazing. Grazing weakened the proved negative effect of species diversity on temporal variation of ANPP (Hautier et al., 2014) and the temporal stabilizing effects of diversity in semi‐arid grasslands. Besides, the compensatory effects between PFGs played an important role in maintaining ecosystem stability. Under extreme droughts, grazing‐induced homogeneity and simplification in community structure may lead to destructive and unrecoverable effects on ecosystem function (Gossner et al., 2016). Although we conducted a six‐year study, this was still not long enough to draw stronger conclusions about the dynamics of ecosystem stability and species diversity in the natural ecosystem. Longer‐term measurements will be required.

The results from the current study confirmed that grazing affected temporal stability not directly by inducing species diversity loss, but by developing the compensatory effects of PFGs, affecting diversity‐dependent species asynchrony and the temporal variation of ANPP. Although our result did not show a grazing‐induced species loss, the species asynchronous responses to environmental change and variability in temporal productivity were all related to species richness and responded strongly to continuous grazing. Although grazing seems not to directly reduce temporal stability and species richness in the current semi‐arid grassland, grazing‐induced homogeneity and simplification in community structure may make grasslands more vulnerable to extreme environment changes. Patterns and thresholds of grazing‐induced changes in ecosystem stability and ecosystem functioning must not be overlooked. Effective grassland management requires a deep understanding of grazing effects on species diversity, PFGs, productivity, ecosystem stability, and their complex interrelationships.

## CONFLICT OF INTEREST

None declared.

## AUTHOR CONTRIBUTIONS

Haiyan Ren, Friedhelm Taube, Yongfei Bai, and Shuijin Hu designed the research. Haiyan Ren performed the research. Haiyan Ren, Claudia Stein, and Yingjun Zhang analyzed the data. Haiyan Ren wrote the manuscript. All authors contributed critically to the drafts and gave final approval for publication.
