# Supplementary material for: Grazing weakens temporal stabilizing effects of diversity in the Eurasian steppe
Source: Ecol Evol. 2017 Nov 26;8(1):231–41. doi: 10.1002/ece3.3669 (PMC5756891; doi:10.1002/ece3.3669)
Supplement: Supplementary file 1 [file ECE3-8-231-s001.docx]

**Table S1** Analysis of variance for the effects of Grazing system (S), Grazing intensity (GI) and Year (Y) on aboveground net primary productivity (ANPP) and richness of plant functional groups. Significant effects of treatments are indicated in bold.

| Variables | Response |  | S | GI | S*GI |  | Y | | Y*S | Y*GI | S*GI*Y |
| --- | --- | --- | --- | --- | --- | --- | --- | --- | --- | --- | --- |
|  | Df. (factor) | | 1 | 6 | 14 |  | 5 | | 5 | 30 | 30 |
|  | Df. (error) | | 14 | | |  | | 70 | | | |
| ANPP (%) | Annual/biennials |  | **6.67*** | 0.82^NS^ | 1.04^NS^ |  | **4.14**** | | **2.37*** | 1.56^NS^ | **1.66^*^** |
|  | Bunchgrasses |  | **10.93**** | 2.05^NS^ | 1.19^NS^ |  | **16.61***** | | 0.73^NS^ | 1.01^NS^ | 1.16^NS^ |
|  | Forbs |  | **6.95*** | 0.27^NS^ | 0.24^NS^ |  | **8.96***** | | 2.01^NS^ | 1.15^NS^ | 1.22^NS^ |
|  | Rhizomatous |  | **18.01***** | 1.91^NS^ | 1.22^NS^ |  | **10.36***** | | 0.26 ^NS^ | 0.80^NS^ | 0.97^NS^ |
| Richness (%) | Annual/biennials |  | 0.05 ^NS^ | 2.25^NS^ | 0.47^NS^ |  | 17.96*** | | **2.45*** | 2.03^NS^ | 0.96^NS^ |
|  | Bunchgrasses |  | 0.41^NS^ | 0.69^NS^ | 0.41^NS^ |  | **24.77***** | | 2.03^NS^ | 1.25^NS^ | 0.66^NS^ |
|  | Forbs |  | 0.88^NS^ | 0.54^NS^ | 0.10^NS^ |  | **23.34***** | | 0.63^NS^ | 1.04^NS^ | 0.89^NS^ |
|  | Rhizomatous |  | 1.94^NS^ | 0.96^NS^ | 0.84^NS^ |  | **35.25***** | | 0.72^NS^ | 1.31^NS^ | 1.22^NS^ |
| ANPP | Annual/biennials |  | **8.87**** | 1.52^NS^ | 1.67^NS^ |  | **5.18**** | | **3.59**** | **2.51**** | **2.61**** |
|  | Bunchgrasses |  | 0.02^NS^ | **4.93**** | 2.69^NS^ |  | **23.03***** | | 0.69^NS^ | 0.96^NS^ | 1.17^NS^ |
|  | Forbs |  | **3.73*** | 0.46^NS^ | 0.37^NS^ |  | **6.72***** | | 1.28^NS^ | 1.26^NS^ | 1.17^NS^ |
|  | Rhizomatous |  | **10.27**** | 1.24^NS^ | 0.93^NS^ |  | **4.98**** | | 0.49^NS^ | 0.75^NS^ | 0.88^NS^ |
| Richness | Annual/biennials |  | 0.19 ^NS^ | **3.01*** | 0.64^NS^ |  | **16.88***** | | **2.44*** | **2.15**** | 0.71^NS^ |
|  | Bunchgrasses |  | 0.04^NS^ | 0.30^NS^ | 1.74^NS^ |  | **6.52***** | | 0.87^NS^ | 0.69^NS^ | 1.47^NS^ |
|  | Forbs |  | 0.22^NS^ | 0.49^NS^ | 0.20^NS^ |  | **28.01***** | | 0.98^NS^ | 1.31^NS^ | 0.87^NS^ |
|  | Rhizomatous |  | 1.29^NS^ | 0.48^NS^ | 0.29^NS^ |  | 0.74^NS^ | | **2.48*** | 0.64^NS^ | 1.57^NS^ |

Note: F-values are shown for each variable followed by their respective significance levels. **P* < 0.05; ***P* < 0.01; ****P* < 0.001; NS, *P* > 0.05. Grazing intensity: 0, non-grazed; 1, 1.5 sheep/ha; 2, 3.0 sheep/ha; 3, 4.5 sheep/ha; 4, 6.0 sheep/ha; 5, 7.5 sheep/ha; 6, 9.0 sheep/ha

**FigureS1** Annual precipitation rates (left y-axis) and annual mean temperature(right y-axis) in Inner Mongolia steppe from 2004-2010.

**Figure S2** The richness percentage of plant functional groups in the whole grassland plant communities during the experimental year from 2005 to 2010 (N = 28) (left) and along a grazing gradient ranging from non-grazed (G0) to heavily grazed (G6) intensity (N = 24) (right).


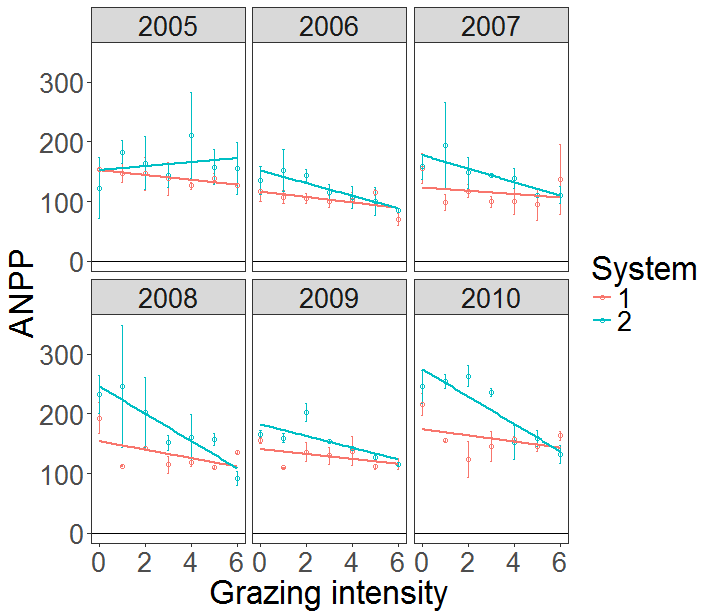


MGSS

TGS

^System^

**Figure S3** Community ANPP (g DM m^-2^) of mixed grazing system (MGS) and traditional grazing system (TGS) along a grazing gradient ranging from non-grazed (G0) to heavily grazed (G6) intensity (N = 24) from the experimental year 2005 to 2010.
